# Ubuntu as a blueprint: learning ethical transdisciplinarity from African indigenous knowledge systems

**DOI:** 10.3389/frma.2026.1783687

**Published:** 2026-02-25

**Authors:** Nceba Gqaleni, Moréniké Oluwátóyìn Foláyan

**Affiliations:** 1 Sub-Discipline of Traditional Medicine, University of KwaZulu-Natal, Durban, South Africa; 2 Africa Health Research Institute, Durban, South Africa; 3 AFRONE Network, Faculty of Dentistry, Alexandria University, Alexandria, Egypt; 4 Oral Health Initiative, Nigerian Institute of Medical Research, Yaba, Lagos, Nigeria; 5 Department of Child Dental Health, Obafemi Awolowo University, Ile-Ife, Nigeria

**Keywords:** African indigenous knowledge, co-creation, decolonization, holism, research ethics, traditional medicine, transdisciplinarity, Ubuntu

## Abstract

This perspective paper posits that the modern global pursuit of transdisciplinarity finds a time-tested blueprint in African Indigenous Knowledge Systems (IKS). It argues that principles such as holism, relationality, and respect, which are intrinsic to philosophies like Ubuntu, are not merely complementary but are essential for conducting ethical, effective, and community-engaged research. The paper offers a critical analysis of the risk of epistemic injustice within contemporary transdisciplinary projects, where ingrained academic power structures can perpetuate extractive and colonial research paradigms. Using the World Health Organization's ethical framework for traditional medicine research as a scaffold, we demonstrate how core tenets of ethical research, including co-creation, fair benefit-sharing, and methodological pluralism, are long-standing, embedded practices within diverse IKS across Africa. We caution against the romanticization or monolithic application of any single IKS, emphasizing the continent's epistemological diversity. By studying and honoring African IKS with nuance and respect, the global research community can move beyond tokenistic participation to achieve genuinely equitable, respectful, and impactful scientific outcomes that are co-created for the common good. This journey requires the decolonization of research methodologies and the critical integration of indigenous paradigms.

## Introduction

The 21st-century research landscape increasingly recognizes transdisciplinarity as essential for tackling complex, wicked problems characterized by conflicting values, knowledge systems, and power dynamics (Bernstein, 2015; Rittel and Webber, 1973; Head, 2008; Head and Alford, 2015). This approach necessitates a profound synthesis of knowledge that transcends academic disciplines to include non-academic and life-world perspectives (Jahn et al., 2012; Lang et al., 2012; Pohl and Hadorn, 2007).

However, the promise of transdisciplinarity is often undermined by the persistent risk of epistemic injustice—the systematic devaluation of Indigenous, local, and experiential epistemologies existing outside dominant Western academic frameworks (Chilisa, 2020; Smith, 2021). This frequently manifests as an extractive paradigm, where communities are treated as data sources rather than genuine partners, relegating community knowledge to the anecdotal and replicating colonial research legacies (Chilisa, 2020; Smith, 2021). The consequences are severe: it dehumanizes communities by reducing them to data points (Kingori, 2015); denies the coherence of indigenous ways of knowing; and leads to cultural alienation, loss of intergenerational knowledge, and internalized inferiority. This fractures relational ontologies like Ubuntu, where identity is co-constituted through connection to community, ancestors, and land (Mbiti, 1990). It also violates autonomy and self-determination in research governance (Garba et al., 2023), ultimately undermining research quality.

For transdisciplinarity to fulfill its ethical and practical promise, it must therefore evolve into a practice of radical reciprocity and epistemic humility, decolonizing the research relationship. This begins with co-defining the problem itself through dialogue, aligning with participatory action research where the community leads (McGrath et al., 2025).

This paper introduces a paradigm-shifting thesis: the principles sought in modern transdisciplinary research have been practiced for generations within African Indigenous Knowledge Systems (IKS). While recognizing that Indigenous epistemologies worldwide share core principles of holism, relationality, and ethical custodianship (O'Regan, 1987; Mahuika, 1998; Vanhulst et al., 2020), our focus on African IKS addresses the urgent need to rectify epistemic injustice in African research. We position Ubuntu, a Nguni (particularly isiZulu) articulation of relational philosophy, not as a monolithic template for all of Africa, but as a richly documented case study of innate transdisciplinarity. Indigenous knowing seamlessly integrated spirituality, ecology, sociology, medicine, and psychology into a coherent whole (Gqaleni et al., 2025; Mkabela, 2005), never confined to a single discipline.

We therefore reflect on the guiding question: Do we need to study our indigenous knowledge generation systems to learn how to conduct respectful and effective transdisciplinary research? We argue yes. Furthermore, authentic community engagement in Africa requires understanding its philosophical foundations. Consequently, the path to authentic transdisciplinarity in Africa lies in learning from the sophisticated, innate transdisciplinarity of African IKS, both as a model and an ethical guide. This paper offers perspectives on the ethical framework of IKS in Africa and suggests ways to engage effectively with this system when designing and conducting research.

## Historical and linguistic context: why isiZulu and Ubuntu?

A critical discussion requires contextualizing our use of isiZulu frameworks and Ubuntu philosophy. Africa is home to immense linguistic and cultural diversity, with over 2,000 languages and a vast array of knowledge systems (Heine and Nurse, 2000). The Bantu migrations, spanning millennia, spread languages and cultural concepts across much of sub-Saharan Africa, leading to shared cosmological themes while fostering distinct regional expressions (Vansina, 1990). We draw significantly on isiZulu conceptual frameworks for several reasons. First, isiZulu is one of South Africa's official languages with a well-documented and academically engaged epistemological corpus, providing a robust entry point for analysis (Gqaleni et al., 2025; Dladla, 2017). Second, the philosophy of Ubuntu (I am because we are), while most famously articulated in Nguni languages, expresses a relational worldview that finds resonance across many African societies, such as *Botho* in Setswana, *Ujamaa* in Swahili, *Omoluabi* in Yoruba, and *Bunengi* in Gikuyu (Gyekye, 1995; Nyerere, 1968; wa Thiong'o, 1986). This makes it a powerful exemplar, not an exclusive model. Third, a focused case study allows for depth in illustrating how a specific IKS embodies transdisciplinary principles, which can then be compared with others.

To counter the risk of presenting Ubuntu as a monolithic stand-in for all African IKS, it is crucial to acknowledge the continent's rich epistemological tapestry. Table 1 provides a non-exhaustive map of this diversity, illustrating that while relationality and holism are widespread themes, their expressions are uniquely shaped by history, ecology, and culture. In West Africa, systems like *Ifá* (Yoruba) constitute a vast corpus of poetic verses, divination, medicine, and ethics, emphasizing balance and destiny (*ayanmo*; Abimbola, 1976). The Akan philosophy of *Sunsum* (spirit) and its ethical system underscore character development and community responsibility (Gyekye, 1995). In East Africa, the concept of Ujamaa (familyhood) in Swahili-speaking regions emphasizes cooperative economics and social collectivism (Nyerere, 1968). Among the Gikuyu, *Bunengi* (virtuous character) guides social relations and governance (wa Thiong'o, 1986). In Southern Africa, alongside Ubuntu, the Kgotla system in Botswana/Tswana culture is a participatory assembly for consensus-based governance and justice (Moumakwa, 2011). In Central and Northern Africa, while often underrepresented in IKS discourse, regions possess deep knowledge systems, from forest cosmologies in the Congo Basin to the ancient Egyptian concept of Ma'at (cosmic order, truth, justice), which governed all aspects of life (Obenga, 2004).

**Table 1:** Comparison of relational principles across African philosophies.

**Philosophy (culture/region)**	**Core principle**	**Key expression in research context**
Ubuntu (Nguni, Southern Africa)	“I am because we are”; personhood through community	Research as a communal project; community as co-investigator
Ujamaa (Swahili, East Africa)	Familyhood, collective economics and responsibility	Research benefits must be collectively owned and reinvested in community welfare
Botho (Setswana, Southern Africa)	Humanness, moral virtue, and interdependence	Researchers must demonstrate moral character (*botho*) to earn legitimacy
Omoluabi (Yoruba, West Africa)	A person of good character, integrity, and wisdom	Research is judged by the ethical character (*iwa*) of the researchers and process
Harambee (Kenya)	“Let us pull together”; communal labor and mutual aid	Research is a collaborative effort where all stakeholders contribute and benefit

We explicitly acknowledge that Ubuntu is not a reflection of IKS. African IKS are plural, dynamic, and context-dependent. This diversity is not a weakness but a strength, offering multiple entry points for ethical engagement. A respectful transdisciplinary approach must begin by identifying and engaging with the specific IKS relevant to the research context, rather than imposing a generic African model. This paper uses Ubuntu as a detailed lens to examine principles that are widely distributed, albeit in culturally specific forms, while actively incorporating examples and concepts from other African regions to counteract generalization.

## Methodological considerations

The analysis and ethical framework proposed in this paper are informed by a participatory and transdisciplinary methodology that itself mirrors the principles of African IKS. The core perspective stems from immersive work within African IKS, particularly drawing on the foundational research presented in *Foundations of African Traditional Medicine: A Nguni Perspective* (Gqaleni et al., 2025). Our methodological approach is guided by two key frameworks. First, we employed an Afrocentric methodology that mandated that the African experience guides all inquiry, recognizing the centrality of spirituality, wholism, and intuition as valid sources of knowledge (Mazama, 2001). It insists that not everything of importance is measurable and that the goal of generated knowledge must be liberating for African people. This methodology ensures the research process is truthful by including knowledge holders not as subjects, but as co-investigators and validators. Second, we utilized Ordinary Language Analysis (Mazama, 2001; Hallen, 2009), which emphasizes understanding the meanings, nuances, and uses of language within its everyday, communal context. This approach prioritizes description and analysis over external criticism, allowing the concepts and logic of IKS, such as *Ubuntu, Isazela*, or *Ukukhunga*, to be understood on their own terms from within the culture.

Operationally, this translated into a community-based participatory research paradigm. Community knowledge holders, including traditional healers (*izanusi/amagqirha*), elders, tribal authorities, and farmers, were integral partners throughout the research process. Their involvement was central to selecting study foci, data collection, analysis, and, crucially, the validation of findings. This approach consciously counters the elitist model of conventional research, which often reduces communities to objects of study (Weinstock, 1971). Instead, it positions the researcher as a facilitator and resource, while the community members are the primary knowers and interpreters of their own reality. Key informants are chosen purposively (Babbie, 2004) to ensure a spectrum of deep experiential knowledge was represented, and their views formed the authoritative core of data interpretation.

## Indigenous knowledge systems in Africa as a model of innate transdisciplinarity

**Figure 1:**
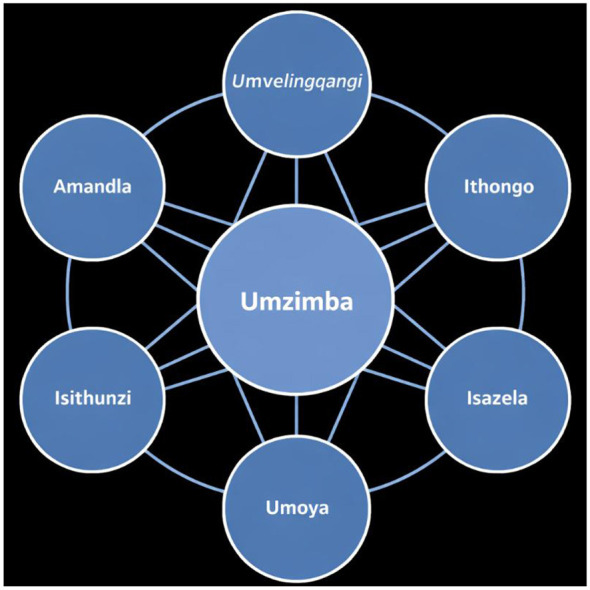
The seven interdependent spheres of existence [Adapted from Gqaleni et al. (2025)].

The depth of African Indigenous Knowledge Systems (IKS) originates from a holistic cosmovision that understands life as a continuous tapestry of connectedness, balance, and reciprocal relationships. This worldview is encapsulated by the philosophy of Ubuntu—“I am because we are”—which posits that individual well-being is inextricably woven into the health of the community, the vitality of the environment, and the harmony of the cosmos (Mbiti, 1990; Gqaleni et al., 2025; Ramose, 1999). This relational ontology is often articulated from a Nguni perspective, specifically drawing on isiZulu conceptual frameworks, providing a refined template for understanding holistic existence. The ontology is structured through seven interdependent spheres (*izizinda*), as illustrated in Figure 1. The term *Isazela* (meaning sphere, realm, or domain of existential awareness) roots the model linguistically and culturally within isiZulu epistemology (Gqaleni et al., 2025). The seven spheres are (1) *Umvelinqangi* (The Creator/Source): The ultimate, singular source and sustaining force of all existence. (2) *Ithongo/Amadlozi* (The Ancestral Realm): The realm of ancestors, who act as custodians of tradition, moral guides, and intermediaries. (3) *Umoya* (The Spiritual Force/Animating Spirit): The pervasive vital force or energy that animates all life and connects the cosmos. (4) *Unembeza* (The Conscience/Inner Moral Compass): The inner faculty of moral discernment and ethical reasoning, linking the psychological to the social and cosmic order. (5) *Umphakathi* (The Community): The realm of human social relations, where personhood (Ubuntu) is realized, and collective well-being is pursued. (6) *Indalo* (The Natural Environment): The living, sacred physical and ecological world, with which humans share a relationship of custodianship. (7) *Umzimba* (The Physical Body): The corporeal vessel, whose health is a manifestation of balance or imbalance across all other spheres.

This integrated practice enables Indigenous Knowledge Systems (IKS) to effectively engage with contemporary transdisciplinary challenges, often termed wicked problems (Gqaleni et al., 2025). Such problems are characterized by interconnected complexities that resist linear solutions, a dynamic reflected in the healer's holistic diagnostic approach. This approach traces root causes across spiritual, social, environmental, and physical dimensions, refusing to isolate issues within a single domain. In African IKS, the traditional healer exemplifies transdisciplinarity by seamlessly integrating cosmology, ancestral communication, botanical expertise, psychological support, and community stewardship into a unified practice (Mbiti, 1990; Gqaleni et al., 2025). The traditional healer (*isangoma* or *inyanga*) operates seamlessly across these spheres: acknowledging the cosmic order (*Umvelinqangi*), consulting ancestral guidance (*Ithongo*), diagnosing spiritual disruptions (*Umoya*), assessing moral-psychological state (*Isazela/Unembeza*), understanding social context (*Umphakathi*), utilizing ecological knowledge (*Indalo*), and treating physical symptoms (*Umzimba*). This is not a mere combination of disciplines, but a coherent, synthesized practice born from a cosmology where such boundaries do not exist (Gqaleni et al., 2025). Figure 1 visually represents the seven interdependent spheres of existence that underpin this worldview, illustrating how health, knowledge, and well-being emerge from balance across all realms.

For transdisciplinary research, this model underscores that challenges cannot be reduced to singular dimensions, such as purely biomedical, but must be engaged across spiritual, social, ecological, and physical domains. Moreover, knowledge is co-constructed through relational exchanges among people, ancestors, and the environment, mirroring the relational ethic essential for equitable research collaborations. Methodological pluralism thus becomes indispensable to honor the integrated reality depicted in this cosmology.

The absence of a definitive stopping rule in wicked problems parallels the healer's enduring responsibility to sustain communal equilibrium. Conflicting values inherent in such problems are mediated through Ubuntu's relational ontology, which fosters consensus and communal harmony (Mbiti, 1990; Mkhize, 2008). Within this framework, research embodies the community itself—a participatory, dynamic process in which community members act as co-investigators, interpreters, and beneficiaries, rather than mere subjects. This reflects the Ubuntu principle that personhood is realized through community (Mbiti, 1990). Consequently, the research process must be collectively designed, governed, and owned to ensure outcomes serve the common good, transcending narrow academic or commercial interests (Hountondji, 1983; Odora Hoppers, 2002). Thus, transdisciplinarity is revealed not as a recent academic innovation but as an enduring, lived practice within IKS, offering a culturally grounded and powerful model for addressing modern complexities (Gqaleni et al., 2025; Kovach, 2009).

## Indigenous practice principles: a cross-cutting ethical framework

Four foundational principles that have historically guided knowledge creation on the continent serve as a robust ethical framework for contemporary transdisciplinary research. These principles are deeply embedded in African IKS and find strong parallels in global Indigenous methodologies (Wilson, 2008; Martin and Mirraboopa, 2003), offering actionable guidance for ethical, equitable, and contextually grounded inquiry.

The first principle, **Respect as Relational Accountability**, entails the active recognition of the intrinsic worth and interconnectedness of all entities—people, ancestors, land, and ecosystems (Mbiti, 1990; Gqaleni et al., 2025; Whyte, 2011). Within IKS, this is practiced through rituals, oral agreements, and custodianship traditions such as *Ukukhunga* (respectful harvesting) in Southern Africa or seeking permission from forest spirits in Central African traditions (van Wyk et al., 1997). In research, this principle challenges extractive paradigms and calls for formalized partnerships where communities are co-investigators, not subjects (Foláyan and Haire, 2023). It aligns with the Māori concept of *whanaungatanga* (relationship-building) and the Native American emphasis on relational accountability (Mkhize, 2008; Smith, 2021).

The second, **Co-Creation as Communal Knowledge Production**, frames knowledge as a collective resource, generated, validated, and sustained across generations (Battiste, 2005; Oguamanam, 2020). This is embodied in practices such as storytelling (Afrofuturist narratives, griot traditions), consensus-based forums like the *Kgotla* (Moumakwa, 2011) or *Gacaca* (Kaufman and Clark, 2009), and intergenerational apprenticeships (Chinweuba and Ezeugwu, 2017). For transdisciplinary research, this ensures that research agendas, methods, and outcomes are culturally rooted and collectively owned, transcending tokenistic engagement. This mirrors the Pacific *Talanoa* method (dialogue for consensus) and Aboriginal *yarning* circles (Vaioleti, 2006; Barlo et al., 2021).

Third, **Benefit-Sharing as Custodianship** positions knowledge and natural resources as sacred trusts held for future generations, obligating fair and equitable sharing of benefits (Garba et al., 2023; Convention on Biological Diversity, 2011). Practices like *Ukukhunga* exemplify this ethos by framing plant use as a partnership rather than ownership. This principle directly informs contemporary ethical standards for intellectual property and benefit-sharing, countering legacies of biopiracy and exploitation, and is enshrined in global agreements like the Nagoya Protocol (Convention on Biological Diversity, 2011).

Finally, **Methodological Pluralism and Holism** affirm that knowledge is validated through complementary ways of knowing—spiritual, ecological, social, and physical (van Wyk, 2015; Hobson et al., 2018). Traditional healers embody this principle by diagnosing illness across multiple realms using diverse methods. In research, it supports integrating qualitative, participatory, and indigenous approaches (e.g., dream interpretation, participatory mapping, seasonal calendars) with conventional scientific methods, enabling more nuanced and context-sensitive inquiry. This aligns with frameworks like Two-Eyed Seeing (Etuaptmumk) from Mi'kmaq territory, which braids Indigenous and Western knowledges (Bartlett et al., 2012).

These principles are lived practices within African communities. Governance structures like the *Kgotla* institutionalize inclusive decision-making (Mbiti, 1990), while rituals embed rigorous, context-aware inquiry into community life (Omodan, 2025). We contend that these indigenous principles constitute a pre-existing, time-tested ethical framework for transdisciplinary research in Africa. They directly counter epistemic injustice by centering community knowledge and authority (Chilisa, 2020; Smith, 2021) and inform concrete research practices such as co-designed agendas, community-led governance with veto power, fair benefit-sharing agreements, and the integration of indigenous and Western methods (Omodan, 2025; Folayan et al., 2019). Moreover, they facilitate the decolonization of research by reshaping physical, procedural, and intellectual spaces to be Afrocentric and inclusive.

## Translating indigenous practice principles into an ethical framework for transdisciplinary research conduct for Africa

The enduring tension between reductionist and holistic paradigms (Fang and Casadevall, 2011) defines much of modern scientific inquiry. While the reductionist approach has yielded profound discoveries, it often fails to capture the emergent properties that arise from dynamic interactions within a whole system. The holistic perspective of IKS and the specificity of reductionist science are not mutually exclusive; rather, they offer complementary insights. Thus, the ethical framework underpinning indigenous knowledge can, and must, be translated into principles to govern robust, modern, transdisciplinary research conducted in Africa. To achieve this, an understanding of the continent's philosophical foundations is necessary (Chilisa, 2020; Ndlovu-Gatsheni, 2018).

### Respect as the foundational ethos

The pursuit of authentic transdisciplinarity in Africa necessitates a foundational ethos of respect, understood as the very bedrock upon which equitable research relationships are built. This ethos emanates from relational ontologies central to African philosophies, which posit that an individual's existence and personhood are co-constituted through interconnectedness (Mbiti, 1990; Checkland et al., 2004). Within this worldview, respect is an active and ongoing practice of acknowledging the intrinsic value and coherence of all elements within this web of life. When translated into the research context, this demands an acknowledgment of African IKS as valid, systematic sciences with their own internal logic, theories, and methodologies, rather than dismissing them as anecdotal or superstitious (Gqaleni et al., 2025; Battiste, 2002). To ignore the ritual, cultural, and ecological contexts in which this knowledge is embedded is to commit an act of epistemic violence, a form of scientific reductionism that strips knowledge of its meaning, power, and vitality (Smith, 2021). Therefore, the ethos of respect mandates a decisive move away from the colonial model of treating communities as mere study subjects and instead, engaging community members and local custodians of knowledge as esteemed colleagues and co-investigators.

Often, the conventional approach to operationalizing respect in practice within the complex and historically fraught terrain of research is to build trust. Trust, in interpersonal and research contexts, is understood as an attitude where one party voluntarily accepts vulnerability based on the positive expectation of the other's goodwill and moral agency (Baier, 1986; Jones, 2012). It is an emotive bond that can facilitate collaboration. In a context marked by historical exploitation and power imbalances, respect cannot be reliably operationalized through the emotive concept of trust, which often places an unfair burden on vulnerable communities. Instead, respect must be structured through formal practices of reflexivity and accountability (Foláyan and Haire, 2023; Omodan, 2025). This shift moves beyond reliance on goodwill to establish verifiable, equitable partnerships.

Translating this principle into practice requires concrete, deliberate actions. First, researchers must engage in the reflexive practice of continuous self-awareness, critically examining their positionality and the potential impact of their work, as a foundational ethical duty (Omodan, 2025). Second, the research relationship must be formalized through collaborative agreements that move beyond informed consent to establish a partnership contract based on accountability. Accountability is defined as being responsible for providing an account for one's actions, conduct, and the discharge of duties (Checkland et al., 2004). Contracts should clearly delineate roles, responsibilities, data ownership, intellectual property rights, and benefit-sharing mechanisms, making all parties answerable to the agreed terms. Third, governance structures must be co-created, ensuring community-led engagement where communities have genuine decision-making power, not just advisory roles. This includes community-led selection of their representatives and granting them equal voting power on project steering committees (Omodan, 2025; Folayan et al., 2019). Finally, there must be pre-defined mechanisms for recourse and sanction, such as formal complaints to institutional review boards that are obligated to act, thereby embedding a liability framework within the partnership (Foláyan and Haire, 2023).

**Co-creation as standard practice**

This respectful engagement, rooted in relationality, naturally evolves into the principle of co-creation as standard practice. Indigenous knowledge is not generated in isolation but is continuously co-created and validated within the community across generations (Battiste, 2002). This operationalizes the Afrocentric view of knowledge as a communal resource (Baier, 1986) and confronts the “empty choice” of coerced participation (Kingori, 2015) by fostering collaborative agency.

Practical implementation requires deliberate structural shifts in power and ownership. Research agendas should be set through joint sessions, not pre-written proposals, ensuring local relevance. The process should be institutionalized through joint steering committees with community-appointed representatives holding equal voting and veto rights (Folayan et al., 2019), and should value indigenous methods (e.g., storytelling, participatory mapping) alongside Western scientific ones as equally rigorous. Finally, data needs to be co-interpreted and outputs co-authored to ensure cultural grounding and community accessibility first.

Rather than imposing Western governance structures, transdisciplinary research in Africa should adopt Indigenous deliberative practices to ensure that research governance is both ethically sound and culturally coherent. The community engagement process should integrate into or align with existing community structures (councils of elders, *Kgotla*, clan meetings). These are community-rooted, ritual-respected gatherings where knowledge custodians (healers, elders, clan leaders) deliberate. Decision-making in IKS is often through dialogue, storytelling, and consensus (Leveridge, 2025). The goal is not majority rule but harmonious agreement that respects all voices. Rather than formal voting, research decisions can be reached through facilitated dialogue and iterative consultation until consensus emerges. This respects relational ontologies and ensures no one is marginalized. Furthermore, in IKS, certain knowledge is protected by custodians who hold the authority to permit or restrict its use. This is a sacred, ethical guardianship. Therefore, research agreements should recognize the moral and spiritual authority of knowledge custodians to say no to certain uses of knowledge or resources. This aligns with practices like *Ukukhunga* and respects the sacredness of Indigenous knowledge.

**Protecting intellectual property and sharing benefits**

The indigenous ethos of custodianship, such as *Ukukhunga*, provides a critical ethical blueprint. This frames knowledge and resources as sacred trusts, not commodities, directly opposing extractive ownership models (Gqaleni et al., 2025). It morally obligates modern research to ensure fair benefit-sharing, acting as an antidote to biopiracy (Convention on Biological Diversity, 2011).

Operationalizing this requires a clear, actionable pathway. Inclusive dialogue needs to be initiated to establish terms, then formalize Prior Informed Consent and Fair and Equitable Benefit-Sharing (ABS) agreements, informed by frameworks like the Nagoya Protocol (Convention on Biological Diversity, 2011). Benefits also need to be negotiated beyond finances to include co-authorship, shared patent rights, capacity building, and support for community-led projects (Onvomaha Tindana et al., 2006). Finally, mechanisms like joint steering committees need to ensure the community has veto power over knowledge and resource use to ensure ongoing accountability (Folayan et al., 2019).

This process is a direct translation of respect into actionable justice in a globalized economy. It ensures that communities are not merely anonymized sources of data or raw materials but are recognized as active, rights-holding partners and the legitimate guardians of the knowledge systems they have safeguarded for centuries (Sors et al., 2023). The framework actualizes relational accountability, ensuring communities are recognized as rights-holding partners and primary beneficiaries, transforming extraction into reciprocity (Hopewell et al., 2025).

**Methodological pluralism and contextualization**

This framework requires methodological pluralism to reflect the anti-reductionist, holistic nature of IKS. Research must employ a suite of contextually defined methods honoring indigenous paradigms (van Wyk, 2015). For example, validating a traditional medicine would test the whole-plant formula as prepared traditionally, respecting the therapeutic synergy of the whole over an isolated molecule (Hobson et al., 2018; Barlo et al., 2021). This pluralism creates a framework for navigating epistemological tensions. A seeming contradiction (e.g., a clinical trial failing to identify a single active compound) becomes a valuable research question. It prompts epistemic humility, inviting investigation into synergistic interactions or non-biochemical pathways, while inviting traditional refinement (Omodan, 2025). Validity is thus judged by the ability to generate understanding that resonates across ways of knowing.

## Decolonization and the Afrocentric research environment

Adopting this framework is an act of decolonization that requires a transformation toward an inclusive, Afrocentric research environment. This shift can be summarized as a move from a colonial reality to an Afrocentric re-imagining, as highlighted in Table 2, developed from the theoretical critiques of Western research structures (Chilisa, 2020; Smith, 2021; Ndlovu-Gatsheni, 2018), insights on re-centering African knowledge (Bartlett et al., 2012), Ubuntu philosophy (Mbiti, 1990; Gqaleni et al., 2025), and integrated with examples from community-based research in Africa that use traditional gathering spaces as sites of knowledge co-creation, and community peer review processes from participatory action research literature (Omodan, 2025; Folayan et al., 2019). The authors' direct involvement in transdisciplinary, community-led research projects further informed the practical dimensions of the table.

**Table 2:** Colonial reality vs. Afrocentric re-imagining in research environments.

**Domain**	**Current (colonial) reality**	**Afrocentric re-imagining**
Physical and epistemic space	Alienating Western architecture (hierarchical labs, lecture halls); knowledge produced about communities in distant institutions	Community knowledge hubs: welcoming spaces (e.g., modeled on the *Indlunkulu, Kgotla*, or village square) with circular seating for dialogue, integrating nature and ritual. Knowledge is co-created *with* and *in* community
Governance and validation	Ethics boards and peer review privilege Western epistemology; community input is advisory	Reconstituted governance: IKS custodians (healers, elders) as full voting members on ethics committees and review panels. Validity affirmed through multi-modal dissemination (e.g., community peer review, feedback sessions, artistic representation)
Human resources and value	No formal career path for community experts; academic rewards based on traditional metrics (publications, grants) alone	Reciprocal capacity building: salaried positions for community scholars-in-residence; revised promotion criteria rewarding co-creation, community impact, and intercultural translation; reciprocal apprenticeships where academics learn from custodians
Funding and ownership	Grants awarded to principal investigators at institutions; data and IP owned by the university/corporation	Community-led resource allocation: funding mechanisms that flow to or are jointly managed by community entities. Data sovereignty agreements; IP governed by ABS agreements that recognize the community as rights-holders

The table serves as a vital bridge between Indigenous wisdom and contemporary research practice, illustrating that studying IKS is essential for redesigning research ecosystems to be equitable, effective, and culturally grounded. It shows how indigenous values translate into concrete research environments. By contrasting colonial reality with Afrocentric re-imagining, the table underscores how IKS challenges and enriches dominant research paradigms. In addition, the table answers the “how” of respectful engagement by proposing actionable reforms in governance, validation, and reward systems. Without this foundational shift, transdisciplinarity risks perpetuating the epistemic injustices it aims to overcome.

A critical step is ensuring community-led selection of representatives, which confronts the colonial legacy of outsiders speaking for communities (Smith, 2021) and counters epistemic tokenism. This upholds self-determination and the Ubuntu principle that personhood is constituted through community relations (Mbiti, 1990). Implementation requires researchers to request humbly that existing community structures appoint representatives through their own protocols, providing support without interference. Representatives must be formally empowered with co-ownership rights and veto power.

Decolonizing transdisciplinary research also requires expanding what is recognized as academically legitimate. Epistemic hegemony excludes Indigenous and local knowledge holders and devalues context-rich, practical wisdom essential for addressing place-based challenges. IKS are lived, performed, and collectively stewarded across generations, deriving power from their applicability, sustainability, and embeddedness within socio-ecological and spiritual contexts (Gqaleni et al., 2025; Mkabela, 2005). Moving toward an Afrocentric research environment, therefore, demands reconstituting knowledge governance by recognizing IKS custodians as legitimate academics and co-validators with equal standing on ethics boards, review panels, and funding committees (Ndlovu-Gatsheni, 2018). It also necessitates valuing multiple forms of evidence and dissemination, such as storytelling, ritual, participatory mapping, and community peer review, as rigorous means of knowledge production and validation (Mkabela, 2005; Battiste, 2002). Academic reward systems must be rewritten to honor co-creation, community impact, and intercultural translation alongside traditional metrics like journal publications (Foláyan and Haire, 2023; Visvanathan, 2009).

This redesign of spaces, structures, and values challenges the university's monopoly on defining knowledge. It calls for research ecosystems where community elders are not visitors but valued co-investigators in a shared project of understanding. Such institutional transformation allows transdisciplinarity to fulfill its promise of epistemic justice, embodying the principle that the well-being of research is inextricably linked to the well-being of the community. This approach does not discard Western science but pluralizes the academy, creating an inclusive epistemological space where different knowledge systems can enter into dialogue, mutual critique, and synergy—aligning with broader calls for epistemic humility and cognitive justice (Folayan et al., 2019; de Sousa Santos, 2016) in addressing complex global challenges.

## Limitations and ethical considerations

We acknowledge the limitations and risks in applying IKS frameworks. First, there is a danger of our romanticizing or essentializing IKS, presenting them as static, perfect, and uniformly harmonious. IKS, like all knowledge systems, can be sites of power dynamics, gender exclusion, or conservatism (Oyěwùmí, 1997). Thus, engagement with our work must be critical and reflexive. Second, there is a risk of over-generalization. Africa is not a country, and Ubuntu is not a universal African philosophy. Researchers must, therefore, invest time in understanding the specific IKS of the community they work with. Third, the instrumentalization of IKS can be a concern when using indigenous concepts to legitimize externally driven research goals rather than genuinely centering them. This may be perceived as a form of epistemic tokenism. Fourth, practical challenges abound, including navigating conflicts between IKS protocols and institutional review board requirements, the time-intensive nature of relational work, and securing funding for non-traditional research structures. Finally, researchers must be wary of speaking for others. Our goal is to promote the creation of platforms for communities to represent their own knowledge systems, guided by the principle of self-determination.

## Conclusion

The ethical framework for engaging with indigenous knowledge custodians for the design and implementation of transdisciplinary research is a gateway to more robust, equitable, and impactful research. First, by reflecting on our indigenous knowledge systems, we find that the answers to many modern research dilemmas have been available to us all along. W**e** must, therefore, study our indigenous knowledge generation systems to learn how to conduct respectful transdisciplinary research in Africa, and build truly respectful, effective, and transformative transdisciplinary frameworks that are by, for, and of Africa. Our proposed ethical framework seeks to replace extraction with reciprocity, tokenism with genuine power-sharing, and epistemic injustice with epistemic humility and cognitive justice. This is a practical ethical imperative that leads to more relevant, sustainable, and innovative solutions. We call for the development and adoption of IKS-informed research ethics guidelines at institutional, national, and continental levels. The widespread application of these principles, adapted to specific contexts, may seem daunting, but the enduring legacy and resilience of indigenous systems prove that it is not only possible but necessary for research that affirms humanity, strengthens identity, and produces knowledge that is both robust and righteous.

## Data Availability

The original contributions presented in the study are included in the article/supplementary material, further inquiries can be directed to the corresponding author.
